# Tracking a Medicine’s Regulatory Risk Management Commitments Provides Better Transparency and Oversight

**DOI:** 10.1007/s43441-025-00825-8

**Published:** 2025-06-20

**Authors:** Simon Ingate, Kate Bendall, Christopher Long, Kevin Fetterman, Marie Liles-Burden, Carmit Strauss

**Affiliations:** 1 Axian Consulting Limited, The Bradfield Centre, 184 Cambridge Science Park, Cambridge, CB2 0GA UK; 2 Feith Systems, 425 Maryland Drive, Fort Washington, PA 19034 USA; 3 Takeda Development Center Americas Inc, 500 Kendall Street, Cambridge, MA 02142 USA

**Keywords:** Risk management commitments, Risk minimisation, Tracking, Additional risk minimisation measures

## Abstract

Pharmaceutical companies are required to provide Risk Management Plans (RMPs) to support the marketing authorisation of medicines. These RMPs contain information on efficacy and safety, outline risks or gaps in information, and detail commitments for post-authorisation pharmacovigilance (PV) studies. These plans aim to enhance understanding of a drug’s benefit-risk profile and information on risk mitigation and labelling strategies to ensure end-users are aware of the risks that are associated with the product. Per defined health authorities trigger, RMPs are updated with new data such as post-authorisation data and expanded product labels (new indications), leading to multiple versions. When specific risks require additional mitigation beyond product labelling, additional risk minimisation materials (aRMMs) may be applied to educate healthcare professionals (HCPs) and patients. In some cases, more stringent controls on drug use may be necessary. In all cases, these risk management commitments must be implemented in applicable countries where the medicine is marketed following local regulatory requirements. Implementing aRMMs necessitates the distribution of core or EU RMPs and aRMMs to local affiliates for localisation, approval by local health authorities, dissemination, and subsequent collection of effectiveness metrics. Managing multiple versions of core and local RMPs, aRMMs, and associated activities becomes a vital data management issue, requiring efficient tracking of the status of each document and locally agreed activities. This article explores developing and deploying a database-driven RMP and aRMM tracking system. It covers the determination of system requirements, the delivery of the system, and the assessment of its impact on the organisation.

## Introduction

Under current regulations from the European Medicines Agency (EMA) [1–3], a risk management plan (RMP) describes a drug’s safety profile and how its efficacy and safety will be monitored and managed post-approval. The European RMP (EU-RMP) [1] summarises the known safety profile of the medicine, highlighting the important identified and potential risks and missing information, as well as the pharmacovigilance (PV) and risk minimisation activities that will be used to manage risks.

The regulator may stipulate a set of commitments that must be implemented to comply with the marketing authorisation of a product. Common RMP commitments include:

- Routine PV [4].
- Post-authorisation safety or efficacy studies [5, 6].
- Routine risk minimisation measures (product labelling, e.g., Summary of Product Characteristics (SmPC) and Patient Information Leaflet (PIL)) [2, 7].
- Additional risk minimisation measures (aRMMs) (ranging from educational materials for healthcare professionals (HCPs) and patients to more complex measures such as restricted or controlled distribution or REMS programs in the USA); [8–10].

**Fig. 1:**
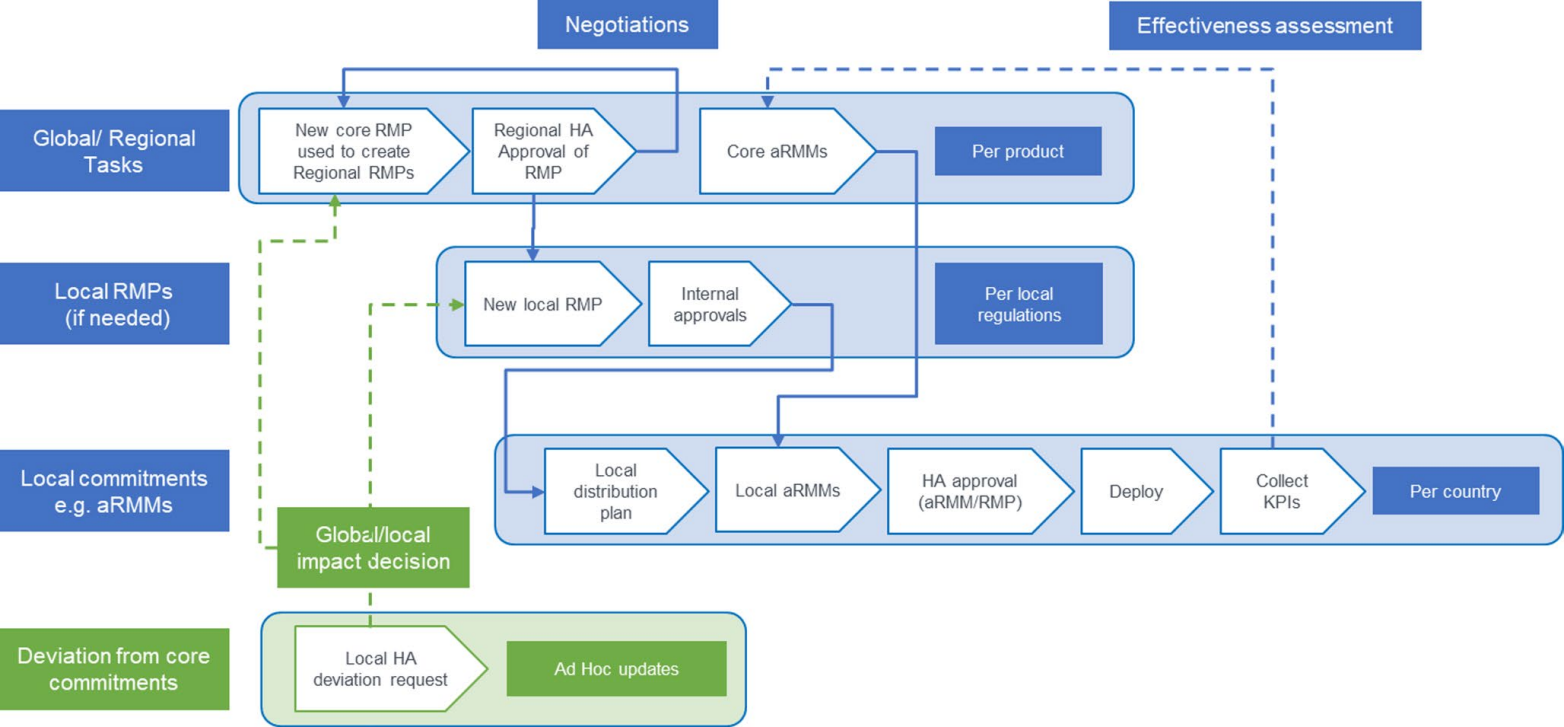
Implementing RMP commitments (here, aRMMs) involves global oversight and local delivery with feedback loops to ensure effective patient safety

Throughout the research on new medicines, development RMPs (DRMPs) or a core RMP can be used to document the investigated product’s emerging safety and risk profile. The RMP should be updated and prepared for submission to the health authorities. RMPs should be updated over time in response to certain triggers such as changes to manufacturing, emerging safety knowledge, efficacy data, and new indications. Companies often create a “core RMP” to document the company’s risk management position and use this as a template to develop local RMPs, which might also include modifications to meet local regulatory requirements. Similarly, if the RMP requires aRMMs, a global organisation may develop “core aRMMs”, which provide a template for the key safety messages in local aRMMs. Figure 1. illustrates how a global pharmaceutical company may manage the development and implementation of the RMP and aRMMs.

Core RMPs are approved internally and converted into the appropriate regional RMPs (such as an EU-RMP) submitted to the regulatory authorities for assessment and approval. When aRMMs are deemed necessary for the safe use of the product, core aRMMs and a global distribution strategy are developed. These global documents are modified to create local aRMMs and distribution plans and are to be submitted to relevant National Competent Authorities (NCA) for approval.

Updates to aRMMs and RMPs may be required if new safety concerns are identified or if new insights on the use of the drug and the application of existing aRMMs/REMS. Local affiliates will prepare such updates where needed, which can occur frequently.

Within the European Union, the EMA’s Good Pharmacovigilance Practice module XVI [8, 9] outlines the types of aRMMs commonly used to mitigate risks and describes the need for effectiveness evaluation. Effectiveness metrics [11, 12] aim to measure the effectiveness of risk mitigation measures. They typically include dissemination outcomes (such as quantities of aRMMs distributed) and knowledge and behavioural outcomes (such as knowledge assessment surveys to assess utility, use, and knowledge or behaviours to evaluate the effectiveness of the measure). Data collected as part of monitoring the progress of commitments is also reported back to regional health authorities in Periodic Safety Update Reports (PSURs) [13] and Periodic Benefit/Risk Evaluation Reports (PBRERs) [14]. Post-authorisation safety studies (PASS) are also used to evaluate the effectiveness of aRMMs and other interventions through user surveys, prescription event monitoring, or different approaches [8, 9].

The updated EU guidance on risk minimisation (part of good pharmacovigilance practices) [8] was published in August 2024. It mandates more regular and rigorous effectiveness evaluations. This will require collecting additional data regarding the distribution, use, and understanding of the content of aRMMs by the intended audience. Other metrics may also be essential to ensure aRMMs are used as intended and to assess if they are fit for purpose or need improvement. Effectiveness data may indicate a need for improvements in the content or distribution of aRMMs.

Global oversight of the progress and success of implementation is critical for all commitments. In addition, local countries should oversee their local commitments, track progress against these, and report on this progress to the global organisation. For an organization with responsibility for multiple medicinal products and where products are marketed in multiple countries, tracking RMPs and aRMMs worldwide may be complex. This article discusses key considerations when planning to implement systems for managing RMP/aRMM commitment tracking. We describe selecting and implementing a tracking system and explore the resulting success of implementation and the benefits delivered.

## Defining the Need for a Tracking System

Companies use various tools to manage and track risk management commitments, which range in sophistication from spreadsheets and e-mail communications to shared areas, collaboration tools, and dedicated purpose-built software solutions. A simple solution may be sufficient for small companies with few products and commitments. However, manual tools are challenging to scale, and these approaches become harder to manage for larger organisations or those with numerous programs and commitments. Companies may need robust approaches to document materials distribution and version management.

Commonly, global safety documentation such as core RMP and aRMM documents are stored in document management systems (which may not be accessible to all stakeholders). These documents may be distributed to local entities via e-mail or spreadsheets to track versions of core RMPs and aRMMs. Similarly, local affiliates may record lists of commitments, RMP and aRMM versions, and information about aRMM dissemination in local and, therefore, isolated spreadsheets. Considering Local Implementation Plans, standard forms describing what has been agreed upon in terms of implementation plans per country, are used and occasionally shared with global colleagues. Many documents are, therefore, shared across companies in ways that are difficult to track.

Manual tracking processes can lead to challenges around consistent delivery, linking to document versions, viewing progress and statuses of implementations, and transparency of information sharing among colleagues across the organisation. Reporting progress from local affiliates to global teams can be complex and disjointed, inconsistencies in the data collected, out-of-date information, infrequent data updates, and data silos could be introduced with higher volumes. Lastly, if implementation tasks are outsourced to third parties, data on progress may not be readily available. Shared areas such as SharePoint sites may increase transparency but the lack of consistent workflow, variable data reporting, missing or erroneous data, and limited reporting capabilities may remain.

Given these challenges, a more sophisticated system is needed to track the process outlined in Fig. 1 and to eliminate or reduce the need for manual information data entry. Business requirements for a dedicated tracking system is outlined in Table 1.

**Table 1:** Business requirements for risk management commitment tracking

Requirement type	Details
Version Control	• Monitor versions of global RMPs and aRMMs and track the version’s differences.
Automation	• Automate key process steps, such as recording the distribution of aRMM templates to local affiliates and their subsequent localisation, approvals, and dissemination.
Deadlines	• Remind users when due dates are approaching and identify missed or late tasks.
Workflows for different tasks	• Provide appropriate workflows and data validation to facilitate data collection, improving transparency and reducing human error.
Reporting and oversight	• Provide oversight on risk management commitments to critical stakeholders (e.g. QPPV) • Deliver management reports to indicate progress and identify issues and process improvements.
Compliance	• Demonstrate compliance and readiness for audit or inspection.

### Selecting a New Tracking System

Organisations where simple tracking approaches become problematic are likely to consider purchasing a purpose-built tracking system (also known as configured off-the-shelf (COTS)) or developing a custom tracking system in-house. Several factors come into play when deciding between a COTS software solution and a custom-developed solution. Custom-developed systems provide more flexibility in design, functionality, and future updates, allowing for highly tailored feature customisations. On the other hand, COTS software is typically faster to implement, has lower upfront costs, receives frequent/periodic updates, and is supported and maintained by software companies catering for many pharmaceutical companies (this user base may identify new requirements benefitting all organisations using these systems).

One available risk management commitment tracking system is the Orbit™ system [15]. This is a validated system allowing users to manage PV and RM commitments, including developing local RMPs from global templates and implementing aRMMs. It provides users with oversight of local variations in safety specifications, commitments, and timelines. It assigns each RMP and aRMM to a “tracker”, a system record type with a unique and configurable workflow that houses each record’s structured data, metadata and documentation.

When using the tracking system, Global users first create “master” or “core” RMP and aRMM trackers and assign local destinations, necessary documentation, and local tasking instructions to these. The system automatically creates and distributes “local” trackers to those countries that must be aware of new commitments so that they can adapt their RMPs and aRMMs locally. Upon receiving the unique tracker for their country, local users follow a predetermined workflow and the timelines provided by Global. This helps ensure consistency in applying global and local rules. The system offers automated notifications and allows users to monitor the progress of implementations in real-time.

In the next section, we describe recommendations we developed from our experience in implementing a new tracking system.

## Defining the Process, Users, and System Requirements

### Reviewing the Current Approach to Tracking

Before implementing a system, it is important to agree and document the goals such a system should help a company achieve (such as improving the workflow, transparency, monitoring, and reporting), as well as to understand the people involved and how they implement and track risk management commitments globally and locally.

**Fig. 2:**
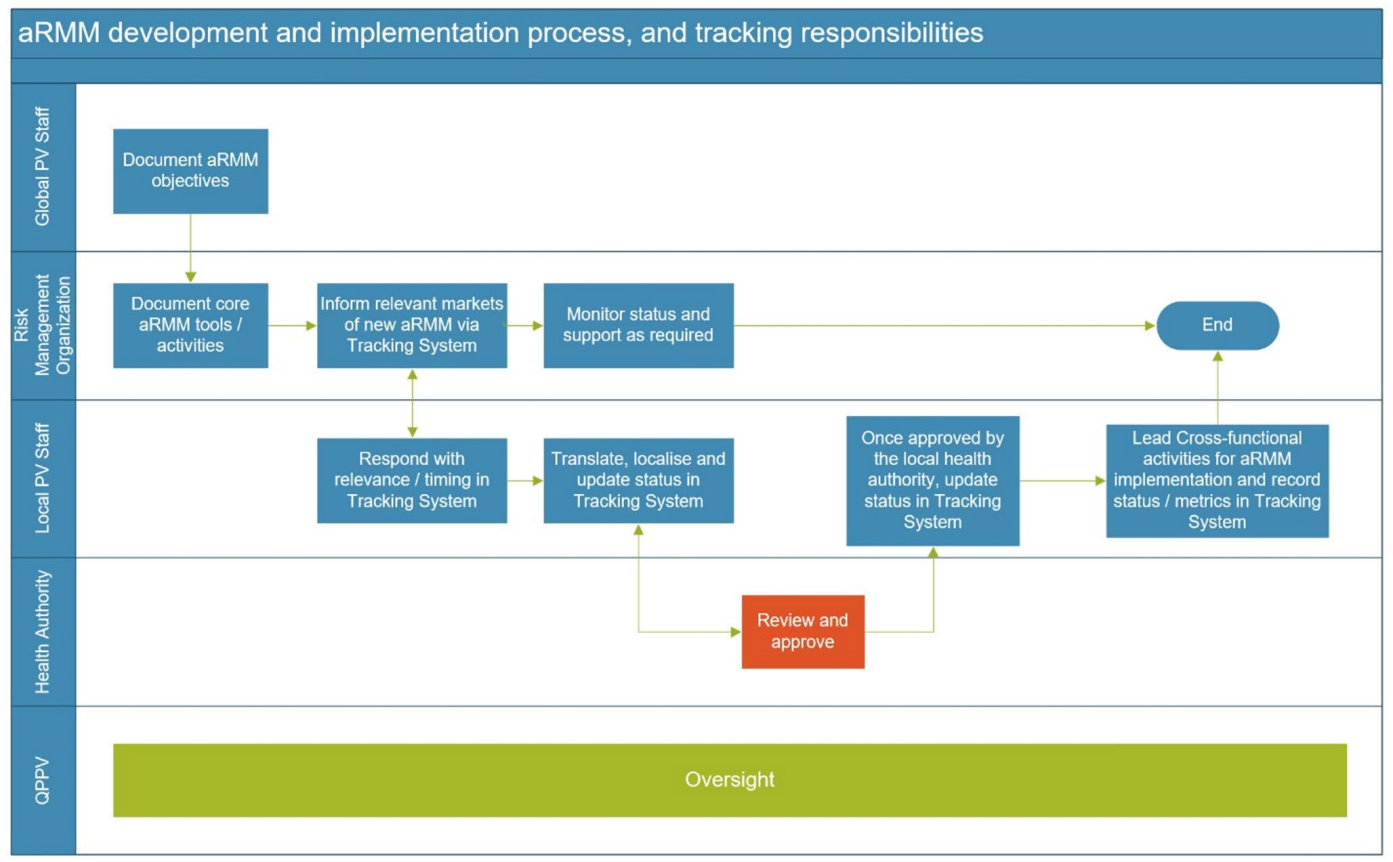
Global to local aRMM development process steps and stakeholders

We worked with a core team in the Global organisation to review the standard operating procedures (SOPs), process maps, and relevant work instructions and to highlight any gaps or aspects that are likely to require revision to meet the anticipated changes in regulatory guidance (Fig. 2) and to realise benefits delivered by the new system. We reviewed local implementation plans to understand the key data elements, and we examined the numbers of RMPs, products and countries where these drugs are marketed to understand the anticipated volume of data.

### Identifying the Requirements of a New Tracking System

We interviewed key stakeholders across the organisation and used their input to define user types (superusers, affiliates, reviewers and system administrators) and process requirements (Table 2). From this information, we have determined the business requirements for the new tracking system. We gathered input from stakeholders regarding the implementation approach. We also set up a group of System Champions (superusers) who were intimately involved in discussions about system requirements, configuration, testing workflows once configured and approving these and tracker contents during the design phase. These Champions then became experts in using the tracking system and critical personnel to educate others.

**Table 2:** Process requirements

Topic	Requirements
Data requirements	• Agree on data to be included in the new tracking system, including data from post-authorisation safety studies (PASS). • Decide how to handle historical data (add to the system or retire). • Ensure the proposed data capture and tracking will meet the needs of effectiveness evaluation.
Tracking needs	• Defined workflows, tracking needs, and automated alerts/notifications required.
Documentation considerations	• Agree SOPs and other process documentation to be updated to reflect the new tracking system.

## Implementing a New Tracking System

### Configuration

The steps followed to configure the new tracking system are shown in Table 3. We then established which products were to be included in the tracking system and the availability of each product in each geographic market. This enabled us to set permissions for dissemination and tracking at regional levels, ensuring consistency and reducing errors in dissemination. For example, if a new set of core aRMMs is developed, they can be distributed to local markets by selecting the “marketed” countries for that product and sending these to all countries simultaneously. As part of this process, local trackers are created.

**Table 3:** System implementation steps

Step	Configuration tasks
User Group set-up	• Define user groups, members of groups and their permissions (process steps each user group can perform)
Define tracker types	• Established trackers for five business process categories: Core RMPs, EU-RMPs, Local RMPs, Core aRMMs, and Local aRMMs.
Configure tracker workflows and contents	• Determined workflows and user permissions (security and access) for each user and tracker type. • Defined and configured the fields, activities, steps, and status names (each tracker has workflow steps and different statuses. • Configured each tracker as follows: a. Set permissions on fields (what is editable, visible, and protected, by whom, at each step in the workflow). b. Defined activities and milestones for each task (e.g., PASS study implementation, aRMM types, or measurement of distribution metrics). c. Defined the status for each task in the workflow (e.g. draft/internally approved/completed)
Configure metrics for reports and build reports	• Defined metrics for reporting, for example, numbers of aRMMs distributed at launch compared with a target number of HCPs/Patients requiring those materials. • Built and configured management reports.

### System Validation and Launch

Implementation and validation were managed in collaboration with the organisation’s IT and validation teams and system users, and a multi-faceted training strategy was developed as part of the launch strategy, Table 4.

**Table 4:** Validation and training tasks

Step	Tasks
System validation	• Documenting and approving the system requirements. • Configuring, testing, and revising system elements as needed in a testing environment. • Validating the system and documenting approvals in tests. • Releasing the approved system as a live version. • Performing user training (see below). • Allowing relevant users to load data. • Providing oversight to Global users who monitored data input and reporting.
Training	• Identifying the applicable audiences for training, • Identifying the information that needed to be imparted, • Identifying the training methods.

In our application of the tracking system, our audience was diverse, including global strategy experts (i.e., risk management experts), global product experts (i.e., safety leads), and local PV specialists. Oversight roles, such as the EU QPPV, were also identified as key stakeholders.

In building the training, we had to consider the key takeaways end-users should know regarding the new system. From there, we created general training for all users and role-based training for specific functions within the workflow (e.g., global safety leads, local PV staff, etc.). General training educated end-users on the system’s overall function, how it related to existing relationships between global and local documents and critical features of the system. Role-based training informed users how to complete system tasks relevant to their job responsibilities (e.g., how a LOC PV staff member would receive and complete a local tracker within the tracking system).

The training conduits were multi-faceted to account for different learning styles and ensure easier system adaptation. These training sessions were available in written format and as live presentations, which allowed for a written reference to drive uniformity and consistency in data entries. An interactive demonstration allowed users to apply system knowledge with examples of practical task completion. Additionally, we created a support structure for staff with more labour-intensive roles within the tracking system by creating times during office hours to ask questions and seek resolution from the global team, IT and system champions.

## Assessing Implementation Success Using the RE-AIM Framework

We used an implementation science framework to evaluate the success of implementing the risk management commitment tracking system. The RE-AIM framework, introduced in 1999, comprises five dimensions: Reach, Effectiveness, Adoption, Implementation, and Maintenance [16, 17]. We describe below how we applied this framework to illustrate the implementation and discuss considerations required in applying the system.

### Reach

Reach refers to the number of individual participants and expands into the participants’ characteristics in the intervention [17]. The end user target audience for the tracking system is especially important to define. Organisations may adopt a centralised operation model where a single point of contact or department is responsible for all activities within the system. Alternatively, they may adopt a more nuanced model which requires multiple functions to complete specific actions within the system. A project’s discovery phase can inform the applicable model and range of individuals invited to participate in the system. In our initial application of the system, we defined participants as end-user PV staff at the global and local levels, yielding over 100 system users, each with specified roles within the system’s workflow.

### Efficacy

Efficacy aims to identify the impact of the intervention, or, in this case, the tracking system’s introduction, on the overall outcome [17]. Impact includes positive and negative outcomes, quality of life, and budgetary considerations. We used key learnings from end-to-end process reviews for existing processes and/or voice of the customer engagements to help define appropriate success measures. To assess functionality, we considered ease of system use and user experience with the system, other ongoing organisational activities, and budgetary guidelines. We also considered enhanced and effective communication and improved access to RMP and aRMM information.

### Adoption

Like the Reach dimension, the Adoption dimension evaluates the number and representativeness of the participants and non-participants. However, Adoption considers the settings and individuals administering the intervention rather than the targets of the intervention [17]. In a regulated organisation where the tracking system is mandatory or built into an organisational process, one can assume 100% adoption. Nevertheless, appropriate adoption of the tracking system may be fostered by the environment made by those administering the intervention. Our application used a multi-layered approach to ensure full adoption by system participants. We identified participants most affected by this system in specific workflows and designated local change champions. These “System Champions” represented a user group within the system and supported end-users within their function. When providing training, we worked internally to minimise friction in the infrastructure that would prevent the system’s adoption.

### Implementation

Implementation describes the efforts used to execute the intervention. Introducing a tracking system requires a concerted implementation plan, including training materials, different presentation formats for different learning styles, and support mechanisms to help new users adopt the system.

We evaluated the system’s effectiveness by surveying local PV personnel. This group of users was selected because local PV staff work more with the tracking system. Their feedback was expected to provide an understanding of sentiment and use of the system from those most impacted by the delivery of the system. We assessed the impact of the system on commitment tracking and communication. The survey design allowed for structured responses to prompts in yes/no format, rating scales and free text responses. The findings were:

- Among 144 local PV staff invited to participate, 39 completed the survey (response rate: 27.1%).
- Respondents included at least one individual from each local regional cluster (range: 1–8 participants per regional cluster, average: 2.79, median: 2).
- Feedback was positive for those who completed the survey, and they recognised the benefits of introducing a tracking system.
  - Approximately 95% of respondents felt that the tracking system improved cross-functional communication and transparency between global and local teams.
  - 87% of respondents agreed that the tracking system had improved RMP tracking.
  - All 39 respondents found it easy to access RMP information from the system, and 93% felt that access to aRMM information had improved due to the implementation of the tracking system.

In addition, since the tracking system allows global users to see all trackers in the system, their status from initial creation to dissemination to local countries, and the progress of local tasks through system queries, reports, and other metrics, Takeda is confident that tracking in our organisation is robust and transparency across the company is improved.

### Maintenance

Maintenance refers to the permanency of behavioural change promoted by the intervention, which is essential in implementing a risk management commitment tracking system. A maintenance plan ensures sustained or improved system effectiveness over time.

The maintenance plan should include accuracy metrics, predefined monitoring checkpoints, and feedback mechanisms. Accuracy metrics define a threshold for measuring the performance of system implementation (e.g., < 1% of key overdue milestones). Maintenance measures we have employed include regular communication with local teams regarding system changes, upcoming and overdue activities, and regular feedback sessions meeting with the “System Champions” and other key stakeholders such as the EU Qualified Person for Pharmacovigilance (EU QPPV). These sessions identify issues with use and opportunities to improve further how we track commitments. Additional reinforcement of system function and compliance supports the overall institutionalisation of the system.

## Conclusions

Large pharmaceutical companies managing numerous products across diverse markets require dynamic and precise control over global and local regulatory risk management commitments. As complexity increases, simple spreadsheets or basic tracking systems become inadequate. Implementing an electronic data tracking solution offers a streamlined, efficient, and consistent approach to worldwide risk management tracking, ensuring robust oversight and compliance across the organisation.

Developing effective tracking systems requires thoughtful planning and alignment with regulatory expectations, organisational needs, structure, and user experience. Successful implementation hinges on understanding user requirements, optimising information dissemination, and ensuring robust metrics to maintain compliance with global regulatory standards. This process demands collaboration across the organisation, thorough system validation to meet business requirements and accurate data management. Post implementation, user training and feedback mechanisms are critical to ensure the system operates efficiently, fosters transparency, and adds value to the organisation. Assessment frameworks such as RE-AIM can further support continuous improvement and enhance the system’s effectiveness.

Implementing the resulting risk management commitment tracking system at Takeda has significantly enhanced the efficiency and consistency of the Takeda PV team’s processes for the automatic rollout of core global RMPs and aRMMs. By streamlining local affiliate tasks and replacing isolated spreadsheets, the system fosters greater transparency and collaboration across the organisation, reduces inconsistencies, and effectively eliminates data silos.

## Data Availability

No datasets were generated or analysed during the current study.
